# Epithelial cell attachment and adhesion protein expression on novel in sol TiO_2_ coated zirconia and titanium alloy surfaces

**DOI:** 10.1002/jbm.b.35111

**Published:** 2022-06-22

**Authors:** Sini Riivari, Elisa Närvä, Ilkka Kangasniemi, Jaana Willberg, Timo Närhi

**Affiliations:** ^1^ Department of Prosthetic Dentistry and Stomatognathic Physiology University of Turku Turku Finland; ^2^ Institute of Biomedicine and Cancer Research Laboratory FICAN West University of Turku Turku Finland; ^3^ ID Creations Turku Finland; ^4^ Department of Oral Pathology and Oral Radiology University of Turku Turku Finland; ^5^ Department of Pathology Turku University Central Hospital Turku Finland

**Keywords:** cell adhesion, dental/endosteal implant, epithelial cells, nanomodified surfaces

## Abstract

An adequate mucosal attachment is important when it comes to preventing peri‐implant inflammation. The aim of this study was to compare epithelial cell adhesion and adhesion protein expression on in sol TiO_2_‐coated and non‐coated zirconia and titanium alloy surfaces. Fifty‐six zirconia and titanium discs were cut, and half of them were coated with bioactive TiO_2_‐coating. To study the epithelial cell attachment, human gingival keratinocytes were cultivated on discs for 1, 3, 6, and 24 h. The cell proliferation was detected by cultivating cells for 1, 3, and 7 days. In addition, the levels of adhesion proteins laminin y2, integrin *α*6, *β*4, vinculin, and paxillin were detected with Western Blot method. Furthermore, high‐resolution imaging of the actin cytoskeleton and focal adhesion proteins was established. Longer‐term cell culture (1–7 days) revealed higher cell numbers on the coated zirconia and titanium discs compared to non‐coated discs. The difference was statistically significant (*p* < .05) after 24 h on coated zirconia and after 3 and 7 days on coated titanium discs compared to non‐coated discs. Clear induction in the protein levels of laminin y2 and integrin *α*6 were detected on both coated samples, meanwhile integrin *β*4 were clearly induced on coated titanium alloy. The microscope evaluation showed significantly increased cell spreading on the coated discs. According to this study, the in sol induced TiO_2_‐coating increases keratinocyte attachment and the expression of adhesion proteins on coated zirconia and titanium in vitro. Consequently, the coating has potential to enhance the mucosal attachment on implant surfaces.

## 1 INTRODUCTION

Dental implants are widely used as a replacement of an extracted tooth. A successful implantation does not only require a proper osseointegration but also an adequate soft tissue attachment against the microbial invasion to the gingival pocket.^1^ Without a proper soft tissue attachment, the oral bacteria are able to invade to the peri‐implant area and cause an inflammatory reaction.^2^ As the inflammation proceeds and the peri‐implant bone starts to resorb, the standing is called peri‐implantitis. Peri‐implantitis is quite a common disease and considerably challenging to treat. In the most advanced cases, it can lead to the loss of implants.^3^ Therefore, prevention of peri‐implant inflammation is crucial.^4^, ^5^ This can be accomplished by ensuring the good soft tissue seal to the implant surface.

Previous studies have demonstrated, that epithelial attachment forms in similar ways to the implant surface as it does to the surface of a natural tooth.^6^ Peri‐implant mucosa consists of connective tissue covered by epithelium. The epithelium attaches to the tooth and the implant surface via hemidesmosomes and extra cellular matrix (ECM) called the internal basal lamina.^7^, ^8^ Laminin‐332, composed of *α*3, *β*3, and *γ*2 chains, is an important adhesion protein localized in the basal lamina.^9^ Another important part in hemidesmosomes is integrin *α*6*β*4, which penetrate through cell membrane and binds to basal lamina protein laminin‐332. Integrin *α*6*β*4 has been proved to be an important factor in epithelial attachment.^10^, ^11^ Previous studies have indicated that the connective tissue under epithelium is not properly attached to the implant or abutment surface but rather the gingival fibers circle the implant abutment parallery, not attaching it directly but forming a connective tissue capsule.^12^ As the epithelial attachment forms the first barrier against bacteria, it plays a crucial role in preventing peri‐implant inflammation. In this study, we focus on to study the epithelial attachment via hemidesmosomes to implant surface.

Means to ensure a proper soft tissue attachment have been studied previously. One option to enhance the mucosal attachment and adhesion protein adsorption is through surface modifications, for example, by using different sol–gel derived coatings.^13^ The benefits of sol–gel derived TiO_2_‐dip coatings are, that it is nonresorbable, thin and easy to produce.^13^ However, the utilization of sol–gel processes is limited, because the flexibility to operate is restricted to only a few kinds of shapes. In fact, more complicated 3D structures require alternative surface treatment methods. In this study, we used modified nanoporous bioactive TiO_2_‐coating that can change surface hydrophilicity, make it biologically active and enhance cell adhesion to the implant surface. In the present study, a novel in sol polycondensation induced TiO_2_‐coating, with a simple coating procedure was evaluated. Instead of dip coating the coatings were produced by direct gelation onto implant surfaces within the sol.

Both zirconia and titanium alloy are frequently used in oral implant abutments. Titanium has been the most used implant material due to its favorable properties, such as biocompatibility and good mechanical strength.^14^ As demands for aesthetic treatment results have increased, zirconia has become an increasingly popular abutment material especially in aesthetic regions. Zirconia fulfills many properties that are expected from implant materials. The good biocompatibility, flexural strength and color that is very similar to natural teeth are ones to name.^15^, ^16^ Above all, the most important thing for good aesthetic result is to prevent the gingival recession from happening, which can be accomplished by ensuring sufficient soft tissue volume and a proper formation of mucosal attachment.^17^

The aim of this study was to compare the effect of TiO_2_‐coating on cell attachment and expression of adhesion proteins on coated and non‐coated titanium and zirconia surfaces. The study was carried out by measuring the amounts of adhered cells, analyzing the cell spreading and the deposition of focal adhesion proteins using confocal microscopy and Western Blot‐method. The hypothesis was, that the TiO_2_‐coating would enhance cell adhesion, proliferation and the expression of adhesion molecules.

## 2 MATERIALS AND METHODS

### 2.1 Sample preparation

Fifty‐six grade 5 zirconia (ZrO_2_ + HfO_2_ + Y_2_O_3_ 99.5% and other oxides 0.5%, Z‐CAD, Metoxit, Switzerland) and 56 grade 5 titanium alloy discs (titanium 90%, vanadium 6%, aluminum 4%) were cut to size of 1 cm^2^ by using a surgical saw (Struers Secotom‐50, Copenhagen, Denmark). The samples were polished by using 1200 grit sandpaper (LaboPol 21, Struers A/S, Rodovre, Denmark). 1200 grit sandpaper results in equivalent surface roughness as typical commercially produced titanium abutments.^18^, ^19^ After the preparation, the zirconia samples were sintered in a ceramic oven at 1400°C for 1 h. Thereafter, both zirconia and titanium discs were washed sonically first with acetone, then with ethanol, for 5 min.

Titanium(IV) isopropoxide, (98 + %, Acros Organics) was dissolved in 95% ethanol. A second solution was made by mixing ethanol, 2‐Ethoxyethanol (99%, Acros Organics) and HCl (Hydrogen chloride, 1 M). This solution was then added to the first solution drop by drop and the resultant solution was stirred vigorously and was left to age at room temperature for 24 h.

Half of the zirconia (*n* = 28) and titanium alloy (*n* = 28) discs were coated with the novel in sol TiO_2_‐polycondensation coating method. Uncoated discs formed the control group. The coating method varied depending on the disc material. The titanium samples were set on a petri dish and covered with TiO_2_ sol wherein the TiOH‐groups attach to the naturally functionally active titanium oxide surface of the substrate via covalent bonds. The samples in sol were kept in the freezer (−18°C) for 2 h. Meanwhile, the zirconia samples needed first to be treated with 2% NaOH to create reactive hydroxyl groups onto the surface. After this, the samples were rinsed with ethanol and dropped to a test‐tube filled with TiO_2_‐sol. The tubes containing the zirconia samples were kept in a cold bath (0°C) for 2 h. After 2 h of TiO_2_‐treatment, the titanium and zirconia samples were washed three times with ethanol and placed in an oven in 500°C degrees for 10 min. Finally, the coated discs were washed with acetone and ethanol, 5 min each.

Scanning electron microscopy (SEM) imaging (2 kV) was done with Apreo S field‐emission SEM (Thermo Scientific, Netherlands) equipped with an Ultim Max energy dispersive x‐ray spectrometer (EDS; Oxford Instruments, UK) to compare the surface topography between coated and non‐coated samples. EDS‐analyze was accomplished on zirconia discs, to confirm the presence of titanium oxide particles on coated zirconia.

### 2.2 Cell cultures

All the samples were sterilized in an autoclave before the cell cultures. In this research, spontaneously immortalized human gingival keratinocytes (GK) were used. The GK were earlier collected from a human gingival biopsy sample.^20^ The cells were blended in keratinocyte‐serum‐free medium (SFM) (Gibco®, Thermo Fisher, USA) and cultured on the samples at a density of 25,000 cells/cm^2^.

#### 2.2.1 Cell adhesion

GK were cultured on zirconia and titanium discs for 1, 3, 6, and 24 h to define the cell adhesion on each surface. The cells were incubated at 37°C in full media and after a certain time period, washed with phosphate‐buffered saline (PBS). The discs were handled with TE‐buffer (10 mmol/L Tris, 1 mmol/L EDTA) and the solution was stored at −70°C. Next, the solution was melted and sonicated for 30 seconds each. Thereafter, the samples were treated with fluorescent nucleic acid stain (Pico‐Green dsDNA, Molecular Probes Europe). One‐hundred microliters of solution from each sample was used to define the fluorescence values using wavelength of 490 and 535 nm (BioTek synergy HT). The total amount of DNA was calculated by comparing the fluorescence values to the standard curve.

#### 2.2.2 Cell proliferation

Cell proliferation was studied by growing GK on the discs for 1, 3, and 7 days after which the specimens were handled with Alamar Blue‐reagent (Thermo Fischer, USA) mixed in SFM. Thereafter, the discs were incubated for 3 h in a CO_2_‐incubator at 37°C degrees. Subsequently, 200 μl of the solution from every specimen was pipetted on a microtiter plate and cell amounts were defined by measuring the absorbance of the samples with the wavelengths of 569 nm and 594 nm (Multiskan FC, Thermo Scientific) and the given values were compared to the standard curve. The relative cell attachment was defined by comparing cell amounts of the coated samples to the values of the control groups.

The samples from Day 1 were fixed with 4% paraformaldehyde for 15 min, after which the samples were washed with PBS and stored at +4°C prior to staining for microscopy.

### 2.3 Western blotting

GK were cultivated for 3 days, washed once with PBS and lysed with TXLB‐buffer [50 mM Tris–HCl, pH 7.5, 150 mM NaCl, 0.5% Triton‐X, 0.5% glycerol, 1% SDS, Complete protease inhibitor (Sigma‐ Aldrich), and phos‐stop tablet (Sigma‐Aldrich)] pre‐warmed to 95°C degrees. The cell lysate was further heated at 95°C for 10 min and samples were stored at −20°C until further treatment. Sample protein content was determined using Protein Assay Reagent (Bio‐Rad) according to the manufacturer's instructions. Equal amounts of protein were mixed with 8xSB (sample buffer) and resolved on Mini Protean TGX Precast SDS‐PAGE Gels (Bio‐Rad), transferred to membrane (Trans‐Blot Turbo Transfer System, Bio‐Rad) and washed twice with mQ (ultrapure water, Milli‐Q) and once with TBST (Tris buffered saline with Tween) prior to blocking in 5% milk in TBST for 1 h. Filters were stained with the following primary antibodies [laminin y2 (1:100, sc‐7652, Santa‐Cruz Biotechnology), integrin *α*6 (1:500, HPA12696, Abcam), integrin *β*4 (1:200, ab110167, Abcam), vinculin (1:1000, v9131, Sigma‐Aldrich), paxillin (1:5000, 612405, BD Biosciences), GAPDH (1:20000, 5G4MaB6C5, Hytest)] diluted in 5% milk overnight at +4°C. The filters were washed three times with TBST, incubated with secondary antibodies [IRDye 680 RD Donkey Anti‐Mouse (926‐68072), IRDye 800 CW Donkey Anti‐Rabbit (926‐32213), IRDye 680 Goat Anti‐Rat (926‐68076), IRDye 800 Donkey Anti‐Goat (926‐32214, 1:5000, LI‐COR Biosciences)] for 1 h at room temperature, washed three times with TBST and imaged with Li‐Cor, Infrared Imager, Odyssey. Three biological replicates were accomplished in Western blotting.

### 2.4 Immunofluorescence staining and confocal microscopy

Fixed samples were permeabilized with 300 μl 0.5% TRITON‐X‐100 in PBS for 15 min. The primary antibodies [laminin y2 (1:100, sc‐7652, Santa‐Cruz Biotechnology), integrin *α*6 (1:100, HPA012696) Bio‐Rad), integrin *β*4 (1:200, ab110167, Abcam), vinculin (1:100, V9131, Sigma‐Aldrich), paxillin (1:500, ab32084, Abcam)] were diluted in 30% horse serum in PBS and incubated overnight. Samples were washed three times with PBS and treated with secondary antibodies [Anti‐Rat (A11077), Anti‐Mouse (A21202), Anti‐Rabbit (A21206) (ThermoFisher Scientific)] and DAPI (nucleus staining, 1:200) and Phalloidin Atto (1:400, Sigma‐Aldrich)] in 30% horse serum in PBS for 1 h at room temperature protected from light. Samples were washed with PBS and mounted onto to microscope glass with Mowiol (Sigma‐Aldrich). The samples were imaged with a spinning disc confocal microscope (63x Zeiss Plan‐Apochromat, Hamamatsu sCMOS Orca Flash4.0, 3i CSU‐W1 Spinning Disk). Cell spreading was defined by measuring the areas of 30 cells from each group. The signal of laminin y2, integrin *α*6 and *β*4 were analyzed by measuring the signal from each staining and normalizing it to the cell amount. Three biological replicates were accomplished in each staining.

### 2.5 Data analyses

The data was analyzed and the graphs were made with GraphPad Prism‐program. The unpaired T‐test and Mann–Whitney U‐test was used in calculating the p‐values. Confocal microscope images and Western Blot results were analyzed by using ImageJ, Fiji‐program.

## 3 RESULTS

### 3.1 SEM analyses

**FIGURE 1:**
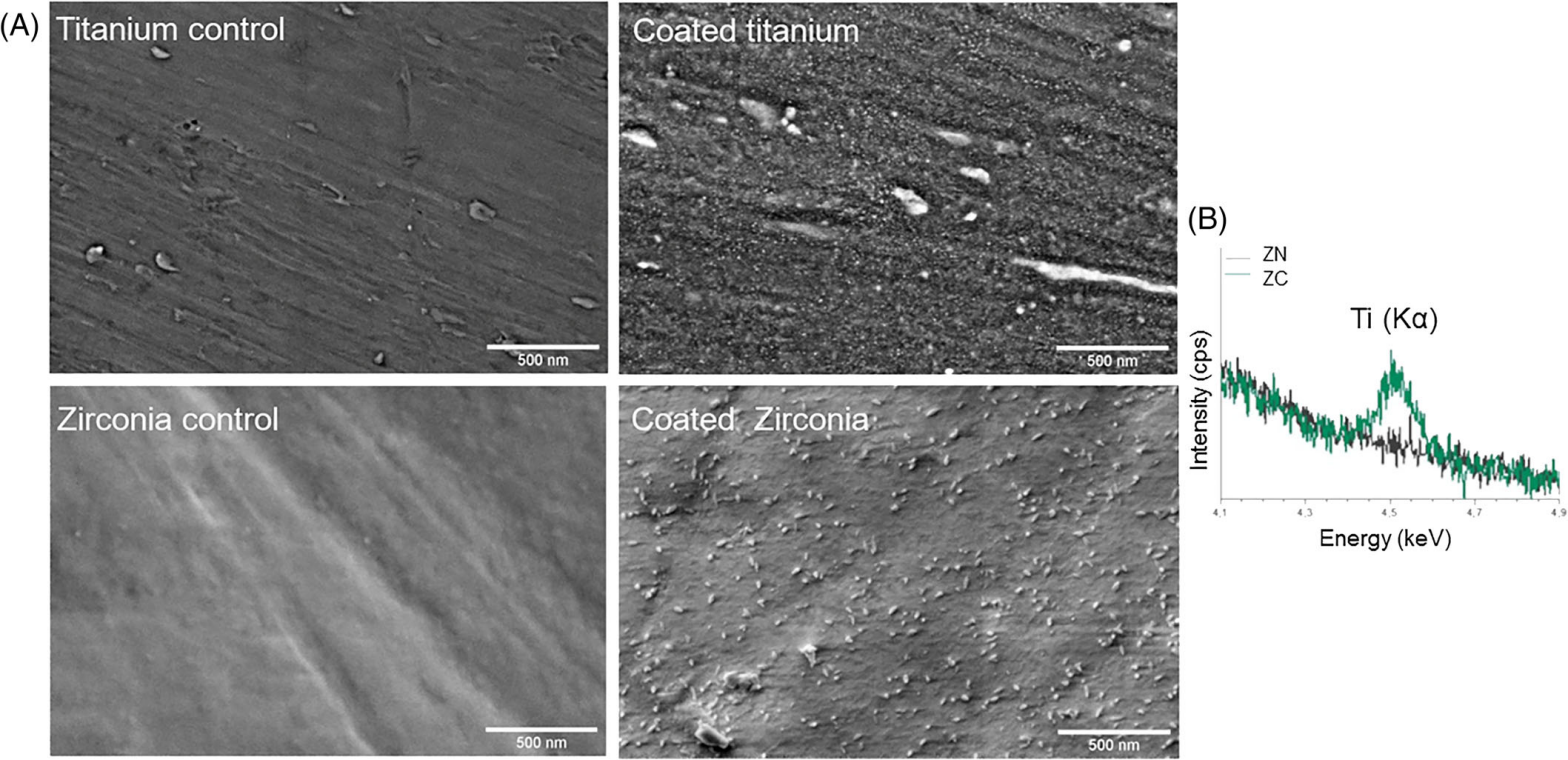
(A) SEM‐images of the surface topography on coated and non‐coated titanium and zirconia. (B) EDS‐analyze indicated the presence of titanium particles on coated zirconia discs while no titanium particles was detected on non‐coated surfaces

The surface topography of coated and non‐coated discs was evaluated using 50,000x magnification. SEM images revealed small, nanostructure particles on coated surfaces (Figure 1A). The particles are similar in size and shape as on titanium surfaces proven to improve cell and tissue adhesion in earlier studies.^21^ EDS‐analyze confirmed the presences of titanium particles on coated zirconia samples, meanwhile non‐coated samples were titanium‐free (Figure 1B).

### 3.2 TiO_2_ coating enhances cell attachment

**FIGURE 2:**
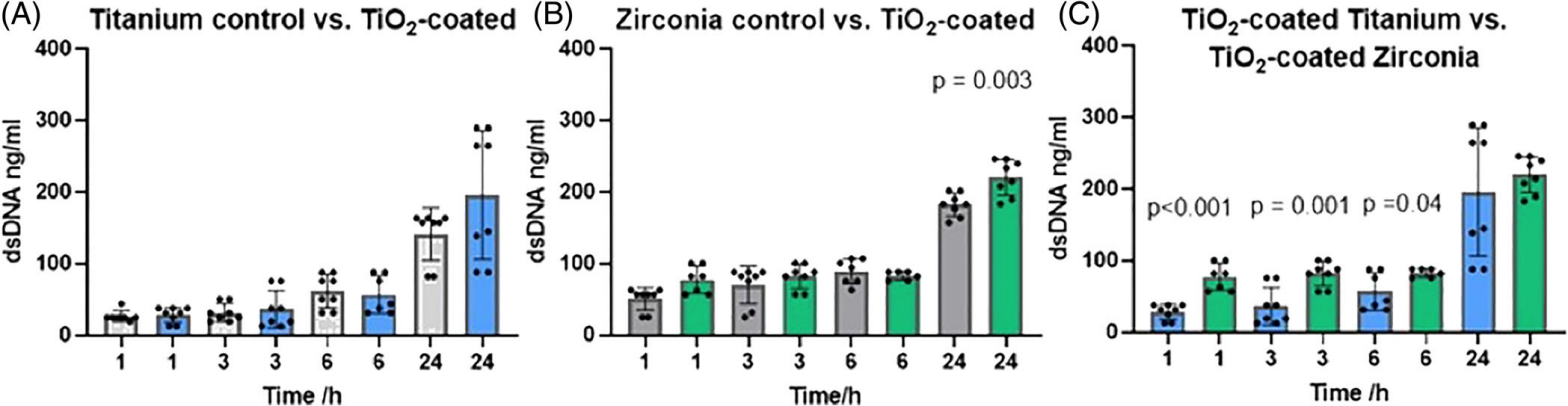
The effect of the titanium/zirconia with or without the TiO_2_‐coating on the number of cells attached during the first 24 h. The amount of cells attached on the different surfaces: (A) Titanium (blue bars), (B) Zirconia (green bars) and (C) comparison of TiO_2_‐coated titanium and zirconia. Gray bars indicate non‐coated surfaces. Cell number was determined based on their DNA content after 1, 3, 6, and 24 h of culture. Data represent mean ± SD. Significant *p*‐values (<.05) are marked in the figures

To determine initial cell adhesion efficiency, short‐time plating assays were performed. The amount of cells attached, determined by DNA amounts, was significantly greater with TiO_2_‐coated zirconia compared to non‐coated zirconia (*p* < .05) after 24 h (Figure 2B). When comparing titanium and zirconia samples, the adhesion was significantly increased on zirconia samples after 1, 3, and 6 h (Figure 2C).

**FIGURE 3:**
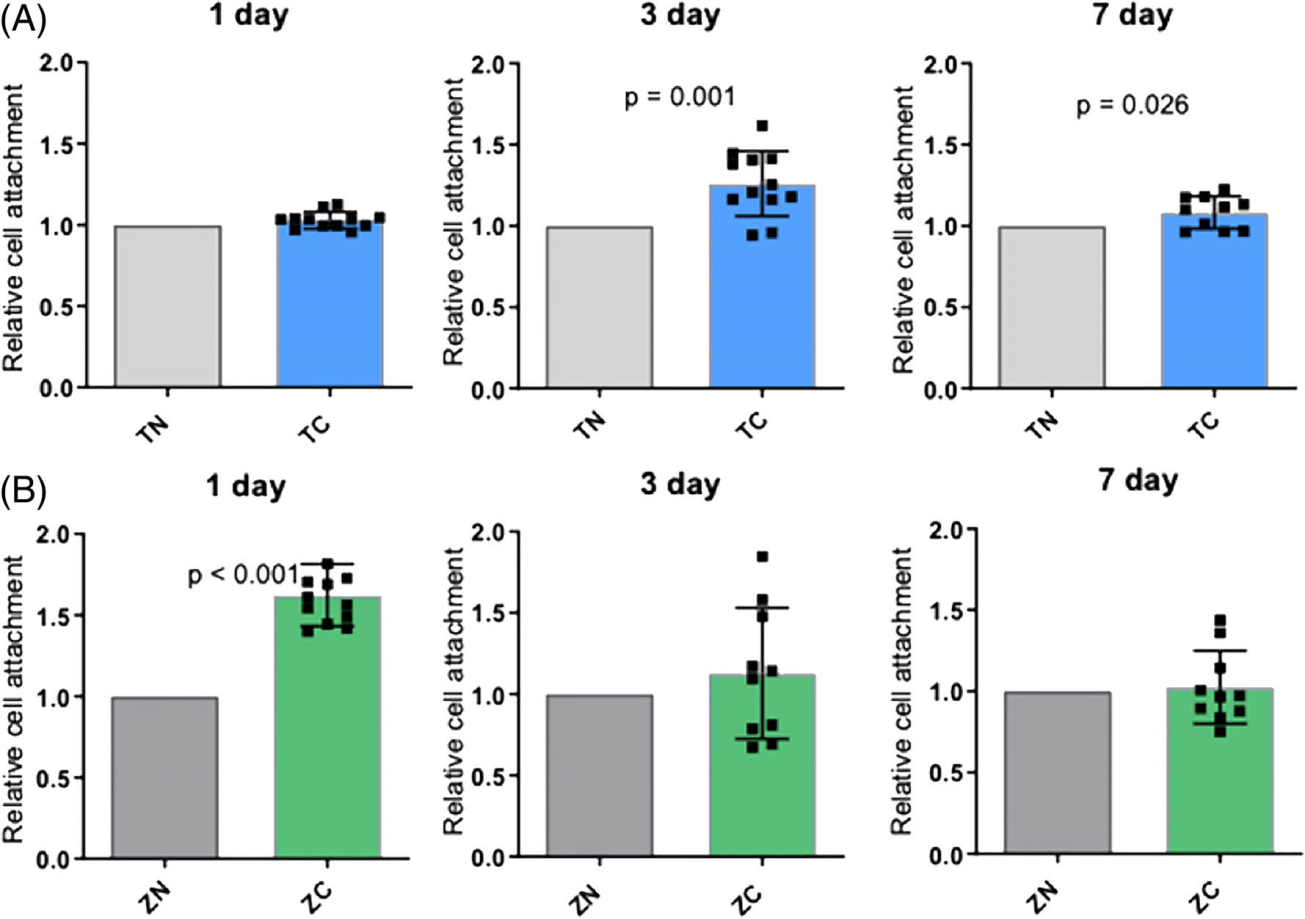
The effect of the titanium/zirconia with or without the TiO_2_‐coating on the relative amount of cells attached and proliferating after 1, 3, and 7 days of culture. Relative cell attachment: (A) titanium and (B) zirconia was defined by giving the control group always the value 1 and dividing the cell amounts of coated samples with the cell amounts of the paired control samples. Data represent mean ± SD. Significant *p*‐values (<.05) are marked in the figures. TC, titanium coated; TN, titanium non‐coated; ZC, zirconia coated; ZN, zirconia non‐coated

In order to study if cell proliferation would be higher on coated surfaces, the cell numbers were measured after 1, 3, and 7 days and relative cell attachment was calculated. A significant difference (*p* < .05) was found between coated and non‐coated zirconia samples after the first day and between coated and non‐coated titanium samples after 3 and 7 days (Figure 3). No significant difference was detected between zirconia and titanium samples.

### 3.3 TiO_2_ coating induces laminin and integrin protein expression

**FIGURE 4:**
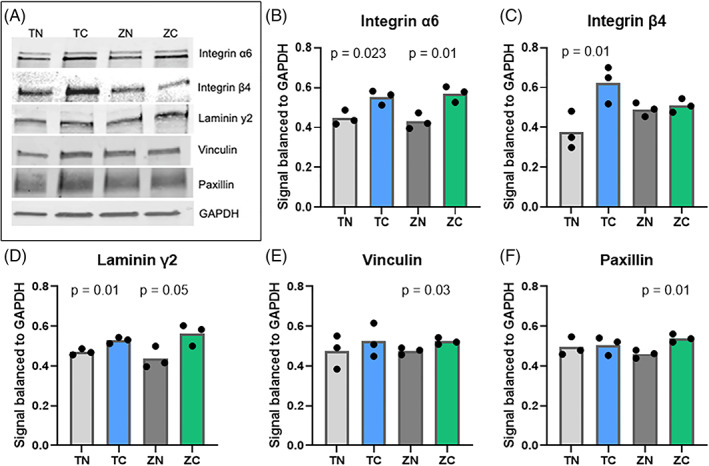
The effect of the titanium/zirconia implant with or without the TiO_2_ coating on the expression of the key adhesion proteins. (A) Western blotting and quantifications of the (B) integrin *α*6, (C) *β*4, (D) laminin *γ*2, (E) vinculin, and (F) paxillin on TiO_2_‐coated and non‐coated zirconia and titanium surfaces from cells grown on the indicated materials for 3 days. Significant *p*‐values (<.05) are marked in the figures

To determine whether the increased cell attachment could be explained by the induction of adhesion protein expression total protein levels were determined. In Western Blot analyses, there was significant induction of laminin y2 and integrin *α*6 on the coated zirconia and titanium samples. The level of integrin *β*4 was also significantly higher on coated titanium samples compared to non‐coated titanium. Meanwhile a clear difference was not found between coated and non‐coated zirconia samples. Paxillin and vinculin levels were significantly higher on the coated zirconia surface compared to the non‐coated samples. Concerning the titanium samples, paxillin and vinculin levels were more equal between coated and non‐coated surfaces (Figure 4).

### 3.4 Confocal microscope analyses

**FIGURE 5:**
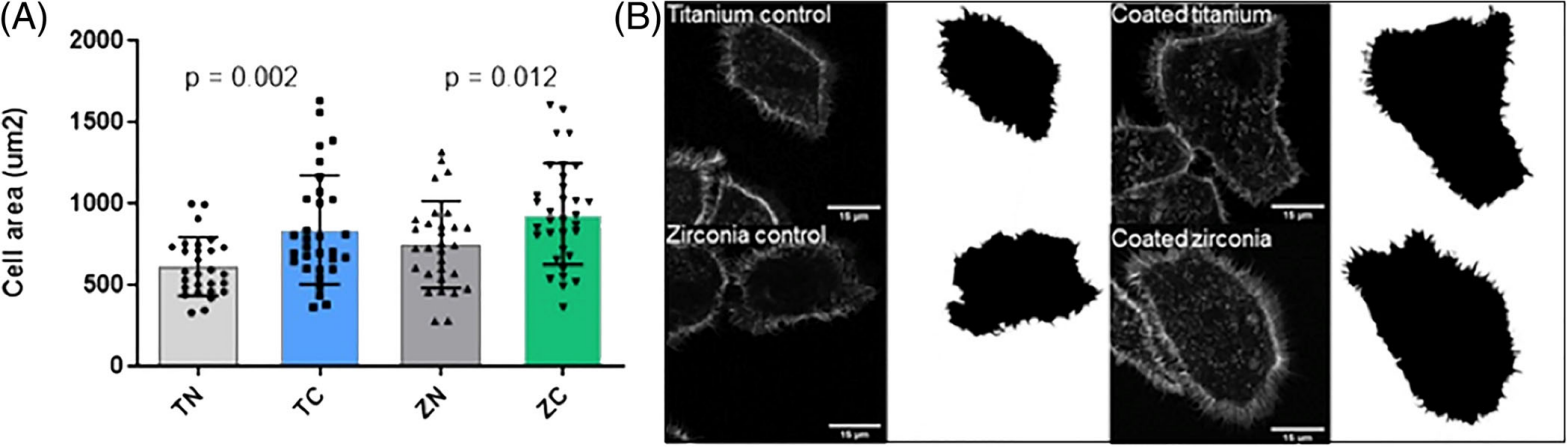
Cell spreading on the titanium/zirconia implants with or without the TiO_2_‐coating after 24 h of cultivation. Shown are quantifications (A) and representative images of cell areas and cells stained with for F‐actin (B). Data represent mean ± SD. TC, titanium coated; TN, titanium non‐coated; ZC, zirconia coated; ZN, zirconia non‐coated

In order to study if increased adhesion protein expression correlated with increased cell spreading, confocal microscope analysis of actin was performed. Cell spreading was significantly higher on both coated zirconia and titanium discs compared to non‐coated controls indicating a faster cell attachment on both coated materials (Figure 5).

**FIGURE 6:**
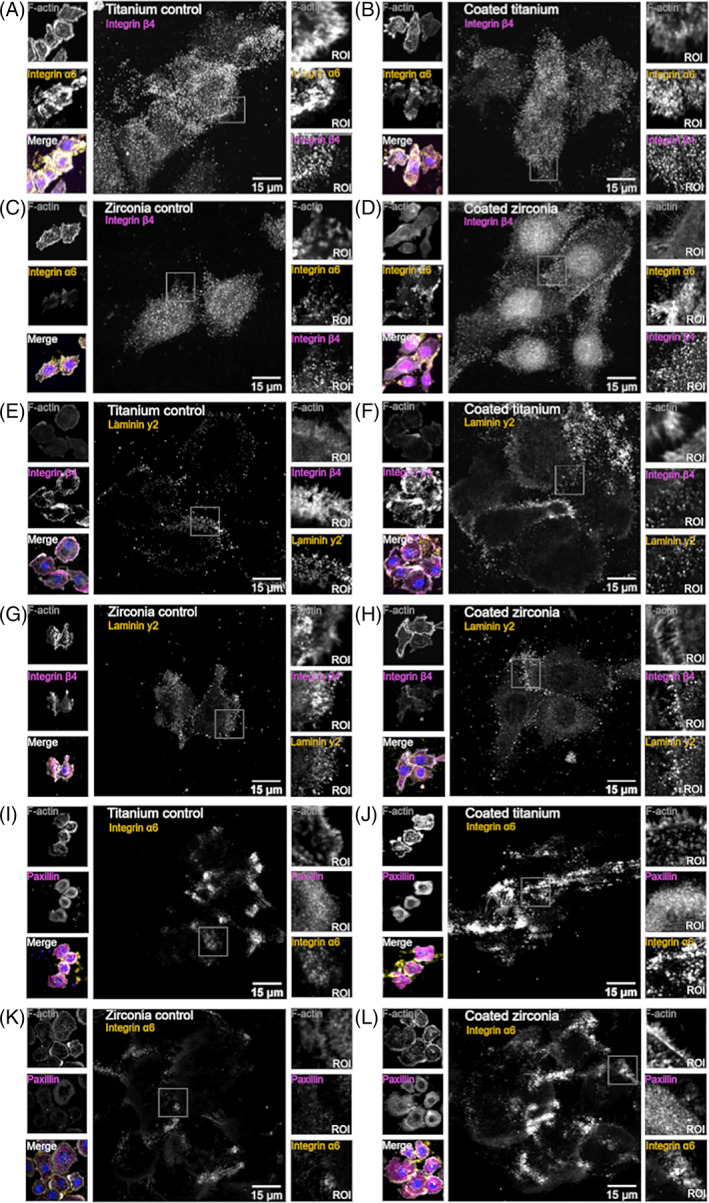
Material coating influences cell adhesion complex formation. Shown are representative confocal microscopy images of the single plane from the cell bottom. The expression of integrin *α*6 (A–D, I–L), *β*4 (A–H), laminin y2 (E–H) and paxillin (I–L) stained together with F‐actin and nucleus (blue). ROI, region of interest (imaged with 3i CSU‐W1 Spinning Disk with 63× Zeiss Plan‐Apochromat objective and Hamamatsu sCMOS Orca Flash4.0 camera)

**FIGURE 7:**
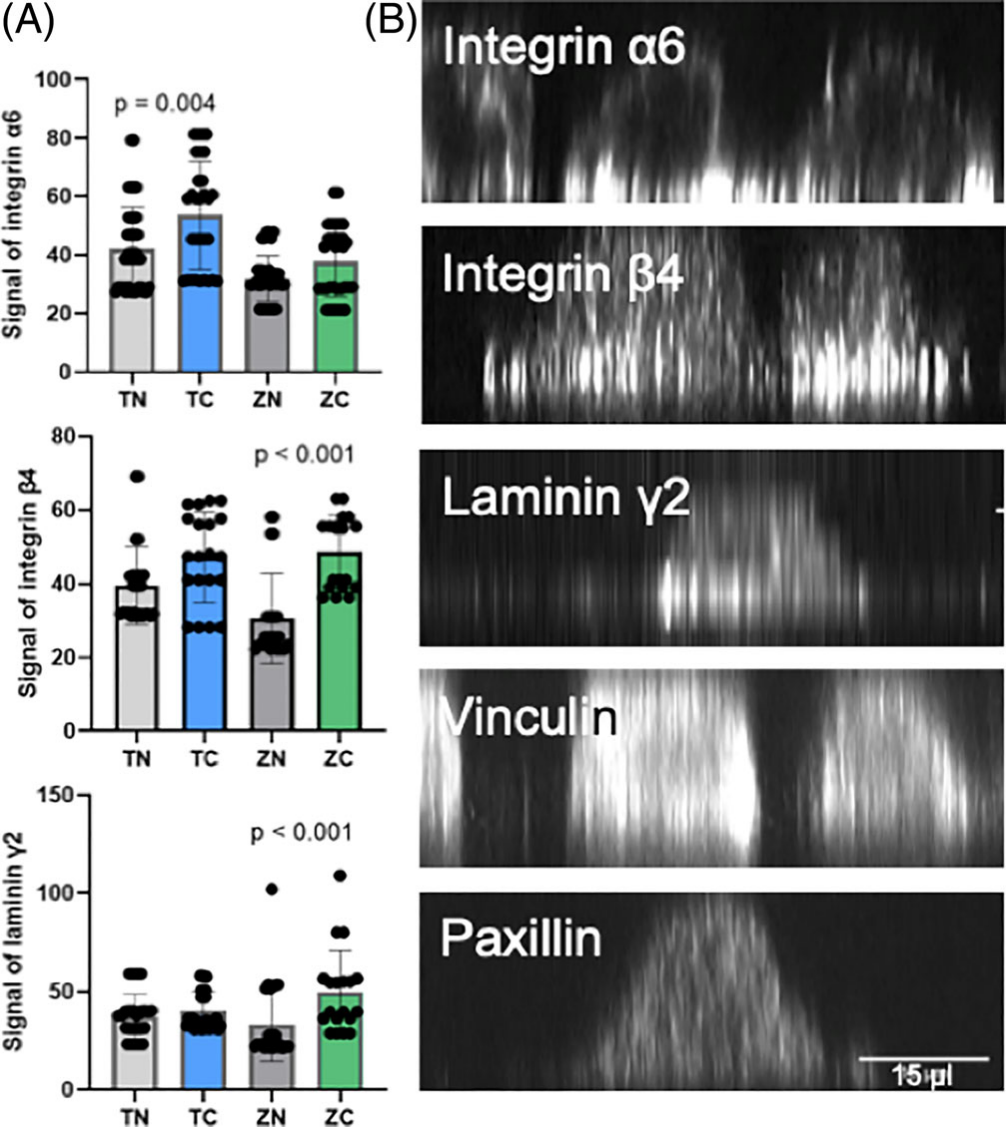
Adhesion molecules laminin *γ*2, integrin *α*6 and *β*4 are mostly located on the bottom plane of the cells in implant adhesions. (A) The signal intensities of laminin *γ*2, integrin *α*6 and *β*4 on the bottom layer per cell in titanium/zirconia surfaces with or without the TiO_2_‐coating. Data represent mean ± SD. (B) The representative images of the orthogonal views of the adhesion protein location in different *z*‐planes (imaged with 3i CSU‐W1 Spinning Disk with 63× Zeiss Plan‐Apochromat objective with Hamamatsu sCMOS Orca Flash4.0 camera)

The adhesion proteins laminin *γ*2, integrin *α*6 and *β*4 were located peripheral at hemidesmosomes indicating that these proteins would participate in epithelial cell adhesion. In turn, the expression of vinculin and paxillin was mostly seen diffusely in the cytoplasm. Figure 6 demonstrates the expression of adhesion proteins on the bottom layer of the cells (Figure 6A–L). The orthogonal view of the samples confirms, that laminin *γ*2, integrin *α*6 and *β*4 had a higher signal on the bottom layer of the cell. Whereas vinculin and paxillin were located more diffusely around the cell (Figure 7B). When measuring the signal volume of laminin *γ*2, integrin *α*6 and *β*4 on the bottom layer of the cells, the volume was higher on the coated samples. The difference was significant concerning integrin *α*6 on titanium samples and integrin *β*4 and laminin *γ*2 on zirconia samples (Figure 7A).

## 4 DISCUSSION

This study revealed that novel in sol induced TiO_2_‐coating enhances epithelial cell adhesion, adhesion molecule expression and cell spreading on both zirconia and titanium surfaces in vitro. Especially laminin y2, integrin *α*6 and *β*4 subunits were clearly induced in keratinocytes cultured on coated samples. This is notable, because these Lm‐332 and integrin *α*6*β*4 are important elements in epithelial attachment via hemidesmosomes.^10^ The study clearly shows that bioactive TiO_2_‐coating can be effective at the molecular level. The results are promising and support the set hypothesis. The study is in line with earlier studies concerning sol–gel derived oven sintered TiO_2_‐coating. Previously, Meretoja et al.^22^ have suggested, that the sol–gel coating is able to facilitate fibroblast adhesion, whereas Rossi et al. demonstrated a clear gingival attachment around tissue level oral implants in vivo.^23^ However, use of sol–gel derived TiO_2_ coatings has been limited due to difficulties in industrial level production of the coatings.

In cultured keratinocytes on both zirconia and titanium samples, the expression of paxillin and vinculin was mostly cytoplasmic, so called non‐focal diffuse punctate distribution. Earlier studies have indicated that paxillin and vinculin are important proteins in focal adhesions.^24^, ^25^ Vinculin has many roles in cell signaling, and it is actin‐binding protein that faces the integrin mediated focal adhesion and this way can affect to the cell attachment.^26^ A result of this study could indicate, that oral keratinocytes attach to the implant surface mainly via hemidesmosomes rather than focal adhesions, as focal adhesion proteins were rarely seen in focal adhesion spots. In Western Blotting, there was a significant difference in vinculin and paxillin levels between coated and non‐coated zirconia samples. However, the microscope images revealed that these proteins would not be in central role in epithelial cell adhesion. Instead, this study shows that laminin *γ*2 and integrin *α*6*β*4 are located in hemidesmosomes, which indicates that these proteins have an important role in cell adhesion to substratum as earlier studies have also stated.^10^, ^27^

This study demonstrates that the in sol induced bioactive TiO_2_‐coating enhances the levels of adhesion proteins and increases cell spreading. The cell spreading is improved supposedly due to the more hydrophilic surface of coated materials. The amount of epithelial cells was higher on the coated samples at the first time points. After 2 days, the cell amounts became quite even. This could mean that coating improves initial cell attachment, but does not influence cell proliferation.

In earlier studies, a sol–gel dip‐coating method has been used.^13^, ^22^, ^23^, ^28^ Dip coating has its challenges when coating more complicated shaped objects. This new “coating made in sol”—technique makes it possible to coat more complex 3D objects. It takes less time and more pieces can be coated in the same time making coating process more efficient. Another potential way to coat implant abutments, is the hydrothermal technique.^29^, ^30^ Earlier studies have also shown that UV treatment is able to make the coating even more hydrophilic and could improve the cell attachment even more.^30^, ^31^ Moreover, the TiO_2_‐coating does not only enhance the surface hydrophilicity but also changes surface topography by making it smooth and nanoporous. The effects of surface treatments on cell attachment have been widely studied and smooth surface has been found to be more favorable for cell attachment.^32^ The TiO_2_‐coating is able to induce calcium phosphate growth on its surface, which has been thought to partly explain favorable soft tissue reactions to TiO_2_‐coated surface.^13^ Although the TiO_2_‐coating is beneficial for cell adhesion, studies have indicated that it does not have the same kind effect to oral bacteria, so the coating does not promote bacterial colonization.^33^

According to this study, in sol induced TiO_2_‐coating functioned in a similar manner on titanium alloy and zirconia surfaces. On both materials, the coating had a positive effect on the cell attachment and protein expression. During the first hours, the adhesion seemed to be faster on the zirconia surface. Nevertheless, the difference between titanium and zirconia became quite even in later time points. Some previous studies have indicated titanium being a better choice when it comes to mucosal attachment. Atsuta et al.^34^ demonstrated epithelial sealing to titanium and zirconia surfaces. In their study, the epithelial bond was weaker and there were only few adhesive structures on the zirconia surface when compared to titanium samples. In the same study, the levels of adhesion proteins integrin *β*4 and plectin were lower on zirconia samples. These results are contradictory, as our study indicated a similar response between zirconia and titanium samples. On the other hand, there are also studies that have suggested zirconia to be the material of choice when a decent soft tissue attachment is wanted. Lee et al.^35^ studied healing patterns between zirconia and titanium. The results indicated a higher expression of adhesion molecules and a more desirable biological width around zirconia abutments. All in all, both zirconia and titanium have their pros and cons. According to this study, there seems no significant difference between these materials when it comes to epithelial cell attachment, but nanoporous TiO_2_‐coating seems to enhance the epithelial cell function on both material surfaces.

## 5 CONCLUSION

To conclude, the novel in sol induced TiO_2_‐coating seems to have favorable effects on epithelial cell adhesion and cell spreading when compared to zirconia and titanium without surface treatment. This could potentially improve the formation of soft tissue attachment to implant or abutment surface. However, in vivo studies are needed to prove the real potential of the coating.

## Data Availability

Original data are available on request from the corresponding author.
